# Social and economic burden of walking and mobility problems in multiple sclerosis

**DOI:** 10.1186/1471-2377-12-94

**Published:** 2012-09-18

**Authors:** James Pike, Edward Jones, Krithika Rajagopalan, James Piercy, Peter Anderson

**Affiliations:** 1 Adelphi Real World, Macclesfield, UK; 2 Biogen Idec, Wellesley, USA

**Keywords:** Multiple sclerosis, Walking, Mobility, Impairment, Burden, Work productivity, Quality of life, Independence

## Abstract

**Background:**

Multiple sclerosis (MS) is a chronic progressive neurological disease and the majority of patients will experience some degree of impaired mobility. We evaluated the prevalence, severity and burden of walking and mobility problems (WMPs) in 5 European countries.

**Methods:**

This was a cross-sectional, patient record-based study involving 340 neurologists who completed detailed patient record forms (PRF) for patients (>18 years) attending their clinic with MS. Patients were also invited to complete a questionnaire (PSC). Information collected included demographics, disease characteristics, work productivity, quality of life (QoL; EuroQol-5D and Hamburg Quality of Life Questionnaire Multiple Sclerosis [HAQUAMS]) and mobility (subjective patient-reported and objectively measured using the timed 25 foot walk test [T25FW]). Relationships between WMPs and disease and other characteristics were examined using Chi square tests. Analysis of variance was used to examine relationships between mobility measures and work productivity.

**Results:**

Records were available for 3572 patients of whom 2171 also completed a PSC. WMPs were regarded as the most bothersome symptom by almost half of patients who responded (43%; 291/683). There was a clear, independent and strong directional relationship between severity of WMPs (subjective and objective) and healthcare resource utilisation. Patients with longer T25FW times (indicating greater walking impairment) were significantly more likely to require additional caregiver support (p < 0.0001), visit a variety of healthcare professionals including their primary care physicians (p = 0.0044) and require more long-term non-disease modifying drugs (p = 0.0001). A similar pattern was observed when subjective reporting of the severity of WMPs was considered. Work productivity was also markedly impacted by the presence of WMPs with fewer patients working full time and a reduction in weekly working hours as T25FW times and the subjective severity of WMPs increased.

**Conclusions:**

In Europe, WMPs in MS represent a considerable personal and social burden both financially and in terms of quality of life. Interventions to improve mobility could have significant benefits for patients and society as a whole.

## Background

Multiple sclerosis (MS) is a chronic inflammatory-demyelinating disease leading to progressive neurological impairment and an array of debilitating symptoms [1,2]. MS affects around 2.5 million people worldwide, is more common among women than men, and is usually diagnosed in adults approximately 30 years old World Health Organization. Multiple Sclerosis International Federation. Atlas: Multiple Sclerosis Resources in the World [3]). MS is often accompanied by functional impairment due to a range of symptoms that contribute to mobility problems including impaired walking ability, loss of arm function, loss of balance, weakness, and muscle spasticity [2,4]. It has been estimated that within 10–15 years of an initial diagnosis of MS, approximately 80% of patients will experience some degree of impaired mobility [4].

Loss of mobility has been shown to impact markedly on employment status, with increasing levels of impairment (as measured using the Expanded Disability Status Scale [EDSS; [5]]) associated with increasing levels of unemployment [6]. Consequently, while the life expectancy of patients diagnosed with MS might be similar to that of the healthy general population [7], their ability to support themselves and any dependents, both financially and physically, can be markedly curtailed by the symptoms of MS. Indeed, loss of productivity either through inability to work, working fewer hours or as a result of having to switch to a less demanding occupation has been estimated to be the single largest contributory factor to the societal burden of the disease [8].

Mobility problems in patients with MS are also strongly related to reduced health-related quality of life (HRQoL) and can profoundly affect the ability of individuals to live independently [6,7,9-14]. Patients with MS can incur considerable costs associated with the provision of mobility aids, home/workplace adaptations, and the requirement for both formal and informal care in order to achieve activities of daily living (ADLs; [7,8,11,14]). The personal physical, emotional and financial burden on informal, unpaid caregivers can also be considerable [15-19]. Caregivers may experience detrimental effects on their own quality of life, particularly when caring for patients with more severe symptoms or with an unstable disease course [15].

Evaluating the severity and impact of mobility problems in MS is essential for the systematic evaluation of the associated disease burden and in the assessment of the effect of interventions to relieve or improve the symptoms of the disease. The evaluation of mobility problems has often relied on subjective reporting by patients or the physician-recorded EDSS which, in its upper end, relies on walking ability (maximum distance) as a measure of mobility problems. Objective, formalized, physician-administered tools for the evaluation and assessment of walking ability in MS are available and include the timed 25-foot walk (T25FW; [20]). Worsening T25FW is associated with worsening neurological functioning and disability [21-23], and has been indirectly correlated to increased care burden and decreased productivity and quality of life [24].

We report here the prevalence, severity and economic burden of walking and mobility problems (WMPs) among patients with MS in 5 European countries (97% of whom were of working age (≤65)). We also relate both self-reported and objectively measured WMPs (using the T25FW) to important aspects of the burden of MS including ability to self-care, healthcare resource utilization, and ability to work, providing a comprehensive picture of the impact of WMPs on disease burden in this setting.

## Methods

### Study design

The MS Disease Specific Programme (DSP^®^) is a cross-sectional real world survey of doctors and their consulting patients conducted in five EU countries between September and November 2009. A total of 340 neurologists in France, Germany, Italy, Spain, and the UK participated in the programme. All patients with MS, as diagnosed by their physician, were eligible for inclusion in the survey. Its real world design ensured collection only of information available to the physician/patient at the time of consultation. Therefore, no tests or investigations were required or conducted for a patient to be included in the study. The full methodology for this survey has been outlined previously [25]. It was performed according to the European Pharmaceutical Market Research Association (EphMRA) Guidelines, and in full accord with HIPAA standards. While ethical approval was not required, each patient provided written consent for anonymous and aggregated reporting of research findings as required by the guidelines.

#### Physician-reported data

During the study period, each participating neurologist completed a detailed patient record form (PRF) for the next 10–12 consecutive patients with MS over the age of 18. Physicians provided information from patient records on healthcare resource utilization including hospitalizations, ER visits, physician consultations, other healthcare professional consultations, and support therapy. Physicians were also asked to provide T25FW data where available but were asked not to conduct the test specifically for the purposes of this study. T25FW data were categorized into 3 ambulation classes relating to completion of the test in <11 seconds, between 11 and 21 seconds, and times in excess of 21 seconds. Given the cross-sectional nature of the data recorded in the Adelphi MS DSP and that worsening walking ability (higher T25FW times) have previously been shown to be associated with worsening neurological functioning and disability [21-23], categorising of the T25FW data in this way allowed the evaluation of the impact of higher T25FW times on the variables included in this report.

#### Patient-reported data

Participating neurologists were also asked to invite patients for whom they completed a PRF to complete a patient self-completion questionnaire (PSC) about their disease, diagnosis, health-related quality of life (HRQoL; as assessed using the EuroQol-5D [EQ-5D] and HAQUAMS), and symptoms including the presence and severity of WMPs. Patients were asked to indicate whether, in their own opinion, their WMPs were mild, moderate, or severe, and to specify their requirement for additional caregiver support (professional or non-professional), home or work modifications, and mobility aids. Patients were also asked about their employment status and the impact of their MS on their ability to work including time lost to work in the previous 12 months due to their MS.

All responses were anonymous to preserve patient confidentiality and to avoid bias at the data collection and analysis phases. The study protocol followed ethical procedures including informed consent of all patients for anonymous and aggregated reporting of research findings based on the questionnaires employed. Patients were instructed by the physician to complete the PSC independently and return it in a sealed envelope, thus ensuring that the physician could not see the responses. Matching the physician and patient responses via patient and physician study numbers allowed the PSC data to be linked with comparable data recorded on the physician-completed PRF. The analyses conducted for the purposes of this paper investigated data from the matched PRF and PSC records.

### Statistical analysis

Chi-squared tests with posthoc Bonferroni-adjusted Chi-squared tests or Fisher’s Exact tests were used to assess the relationship between the T25FW and physician- and patient-reported disease characteristics and healthcare resource utilization. Analysis of variance (ANOVA) with Bonferroni corrected t-tests was used to assess annual days off work. All analyses were carried out using STATA 10.1 (StataCorp 2007. Stata Statistical Software: Release 10. College Station, TX: StataCorp LP).

## Results

Physician-recorded patient data (PRFs) were available for 3572 patients, of whom 2171 (60.8%) completed a patient-recorded questionnaire (PSC). T25FW data were available for 184 patients (5.1%). Table 1 details the demographics and disease characteristics for the total patient population (N = 3572).

**Table 1:** Demographics and disease characteristics

**Parameter**	**N = 3572**
Gender, %	
Male	36
Female	64
Mean age, years	40.6
Mean body mass index, kg/m^2^	23.9
Mean number of years since diagnosis of MS	4.6
Has a caregiver, %	32
Disease Progression, %	
Stable	54
Deteriorating slowly	40
Deteriorating rapidly	6

### Burden of WMPs in MS

Of the patients who provided information about the presence and severity of WMPs (N = 2111), 1342 (64%) reported no WMPs, 271 (13%) reported having mild WMPs, 314 (15%) reported having moderate WMPs, and 184 (9%) indicated that they considered their WMPs to be severe.

### ‘Bothersomeness’ of WMPs in MS

Among the patients who responded regarding their most bothersome symptom (N = 683), 291(43%) patients considered this to be their WMPs. Of these 291 patients, 78 (27%) reported having mild WMPs, 122 (42%) reported having moderate WMPs and 91 (31%) reported having severe WMPs.

### Requirement for care and mobility assistance

**Figure 1:**
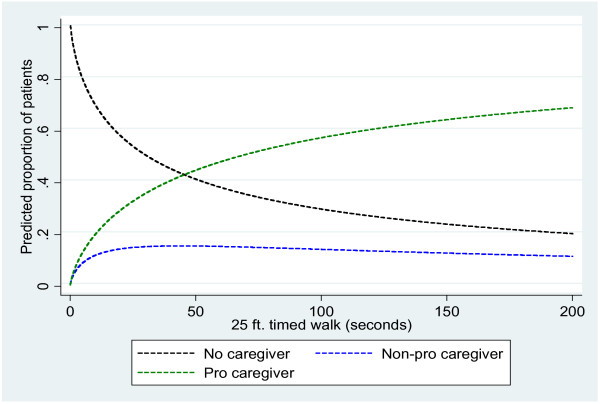
Relationship between increased T25FW times and the need for patient caregiver assistance.

Among patients with both T25FW and caregiver requirement data (N = 183), ordered logistic regression analysis showed a significant association between T25FW and overall (professional and non-professional) caregiver requirement (P < 0.0001) with patients with longer T25FW times being more likely to require both formal and informal care (Figure 1).

Among patients who provided information about the presence and severity of WMPs and healthcare resource utilization (N = 2063), need for mobility aids also rose with increased WMPs. The proportion of patients requiring a walking frame increased from 3% for patients with no WMPs to 6% (mild), 13% (moderate) and 24% (severe) (P < 0.0001) (P < 0.0001). Among patients with no or only mild WMPs, 13% required a cane or walking stick compared with 39% of patients with moderate WMPs and 28% of patients with severe WMPs (P < 0.0001). However 58% of patients with severe WMPs require the use of a wheelchair compared with only 6% of those with no WMPs, 7% with mild WMPs and 11% with moderate WMPs (P < 0.0001).

### Healthcare resource utilization

Among patients with both T25FW and healthcare resource utilization data (N = 184), statistically significant increases in overall resource utilization among patients were observed as timed-walk duration increased (Table 2). Specifically, primary care consultations (p = 0.0044), urologist visits (P = 0.006), time spent with occupational therapists P = 0.0104, and the number of long-term non-disease modifying drugs used increased significantly with increased duration of the T25FW (P = 0.0001). No significant differences were observed in the frequency and length of hospital stay with increased duration of the T25FW. The pattern for ER visits in the previous 12 months was more complex. There was no difference in the number of annual visit for patients in the lowest and highest T25FW time groups (0.32 and 0.31 visits in the <11and >21 seconds, respectively). However, the number of annual ER visits was markedly lower for those in the 11–20.99 second group (0.15 visits).

**Table 2:** Summary of healthcare resource utilization by T25FW category and patient self-reported WMP presence and severity

**Resource (mean [SD] use in last 12 months)**	**Patient-self-reported presence and severity of WMPs**					**T25FW (seconds)**			
	**None**	**Mild**	**Moderate**	**Severe**	**P-value no WMPs vs any WMPs**	**<10.99**	**11–20.99**	**>21**	**P-value across groups**
ER visits	0.23 [0.61]	0.24 [0.63]	0.21 [0.68]	0.45 [1.09]	0.014	0.32 [0.53]	0.15 [0.41]	0.31 [0.59]	0.1592
Physician visits									
Responding neurologist	3.23 [2.55]	3.93 [4.51]	3.76 [3.58]	4.13 [6.39]	0.003	5.07 [9.76]	3.68 [3.86]	3.60 [2.36]	0.3694
Primary care provider	1.71 [2.62]	2.01 [3.97]	1.86 [3.11]	2.86 [4.23]	< 0.0001	1.37 [2.20]	2.84 [4.33]	3.13 [3.12]	0.0044
Other neurologist	0.59 [1.09]	0.51 [1.11]	0.81 [1.91]	0.80 [1.38]	0.0045	0.80 [1.14]	0.61 [0.96]	0.54 [0.94]	0.3341
MS specialist	0.30 [1.09]	0.26 [0.75]	0.36 [1.24]	0.52 [1.59]	0.0633	0.75 [1.18]	0.47 [0.95]	0.42 [1.00]	0.1718
MS nurse	0.36 [2.05]	0.42 [1.99]	0.45 [1.91]	0.42 [1.83]	0.8858	0.33 [0.74]	0.47 [1.26]	0.35 [1.12]	0.7124
Internist	0.06 [0.40]	0.09 [0.52]	0.08 [0.42]	0.23 [1.11]	0.0006	0.27 [0.98]	0.11 [0.67]	0.10 [0.50]	0.3628
ER physician	0.03 [0.36]	0.29 [3.11]	0.05 [0.58]	0.05 [0.34]	0.0116	0.01 [0.12]	0.02 [0.13]	0.00 [0.00]	0.6577
Physiotherapist	1.97 [10.45]	4.74 [15.54]	6.88 [18.35]	9.89 25.52]	< 0.0001	3.63 [14.57]	10.26 [31.77]	6.04 [15.82]	0.22
Ophthalmologist	0.19 [0.58]	0.21 [0.82]	0.10 [0.42]	0.15 [0.57]	0.0579	0.23 [0.65]	0.21 [0.65]	0.10 [0.36]	0.4269
Urologist	0.12 [0.47]	0.18 [0.85]	0.30 [0.86]	0.57 [1.37]	< 0.0001	0.12 [0.54]	0.18 [0.43]	0.50 [1.00]	0.006
Gastroenterologist	0.01 [0.13]	0.03 [0.49]	0.02 [0.15]	0.03 [0.19]	0.3492	0.01 [0.12]	0.04 [0.19]	0.08 [0.33]	0.2716
Psychiatrist	0.19 [1.65]	0.28 [1.70]	0.28 [1.27]	0.69 [4.05]	0.0595	0.35 [1.79]	0.25 [0.63]	0.42 [1.23]	0.7918
Other physician	0.04 [0.31]	0.28 [3.19]	0.10 [0.51]	0.17 [0.70]	0.0222	0.05 [0.28]	0.04 [0.19]	0.08 [0.44]	0.7806
Occupational therapist	0.01 [0.22]	0.06 [0.51]	0.20 [1.09]	1.11 [2.47]	< 0.0001	0.09 [0.66]	0.00 [0.00]	0.77 [2.11]	0.0104
Hospital visits due to MS in last 12 months	0.93 [1.26]	1.11 [1.55]	1.17 [1.64]	1.76 [2.72]	< 0.0001	1.04 [0.81]	0.93 [1.28]	0.70 [0.74]	0.1561
Number of long-term non-DMDs	0.54 [0.78]	0.82 [0.92]	1.23 [1.12]	1.72 [1.18]	<0.0001	0.57 [0.76]	1.21 [0.86]	1.12 [1.02]	0.0001

DMDs, disease-modifying drugs; ER, Emergency Room; MS, multiple sclerosis; SD, standard deviation; T25FW, timed 25-foot walk test; WMPs, walking and mobility problems.

Among patients who provided information about the presence and severity of WMPs and healthcare resource utilization, the presence of patient-reported WMPs correlated significantly with almost all health care resource use parameters measured as part of this study including mean annual ER visits (P = 0.0014), primary care consultations (P < 0.0001), neurologist (P = 0.0045), internist (P = 0.0006) and urologist (P < 0.0001) consultations, as well as time spent with other health care providers, the number of long-term non-disease modifying drugs (P < 0.0001), and hospitalizations in the last 12 months (P < 0.0001) (Table 2).

### Ability to work

Among patients with T25FW and employment data (N = 184), the number of patients working full time decreased as T25FW time increased (p < 0.0001 across the three groups; Table 3). The greatest decrease compared with those in the <11 second category was among those in the 11–21 second category 56.0% vs 12.7%, respectively). The proportion of patients working part time increased compared to the <11 second group (12%) to approximately 3-fold in the 11–21 second category (38.2%), and approximately 2-fold in the >21 seconds category (19.2%) (p = 0.0015 across the three groups). A greater proportion of patients reported a reduction in their weekly work-hours as the duration of the T25FW increased although the differences across the three groups did not reach statistical significance (P = 0.0527).

**Table 3:** Summary of work activity by T25FW category and patient self-reported WMP presence and severity

**Work activity**	**Patient-self-reported presence and severity of WMPs**					**T25FW (seconds)**			
	**None**	**Mild**	**Moderate**	**Severe**	**P-value no WMPs vs any WMPs**	**<10.99**	**11–20.99**	**>21**	**P-value across groups**
Full time, % respondents	46.55	34.70	19.03	9.34	<0.0001	56.00	12.73	38.46	<0.0001
Part time, % respondents	15.77	22.76	17.42	8.24	0.0006	12.00	38.18	19.23	0.0015
Reduced weekly working hours due to MS, % respondents	11.54	25.68	22.37	21.59	<0.0001	22.00	32.50	25.58	0.527
Stopped work due to MS, % respondents	7.16	11.67	28.95	38.07	<0.0001	14.00	15.00	13.95	0.9881
Annual days off work due to MS, mean [SD]	20.86 [43.45]	32.06 [52.39]	55.02 [106.69]	74.14 [120.59]	<0.0001	23.81 [28.85]	41.00 [91.33]	75.16 [182.03]	0.2273

Of the patients who provided information about the presence and severity of WMPs and work activity, statistically significant reductions in all measures of work activity (full-time, part-time, annual days off work, reduced weekly work-hours, and stopping work) were reported with the presence and increasing severity of WMPs (Table 3).

### Representativeness of the cohorts

Additional analyses were undertaken in order to determine whether there were any significant differences between the various cohorts in that were compared in the analyses described above (Table 4).

**Table 4:** Representativeness of the PSC vs non-PSC and the T25FW and non-T25FW cohorts

**Characteristic**	**PSC**	**Non-PSC**	**P-value**	**T25FW**	**Non-25FW**	**P-value**
Gender, %	N = 2169	N = 1397	0.0384	N = 184	N = 3382	0.8527
Male	38	34		36	37	
Female	62	66		64	63	
Employment type, %	N = 1048	N = 593	0.0004	N = 98	N = 1543	0.2362
Manual skilled	25	30		22	27	
Manual unskilled	15	19		12	17	
Non-Manual	60	51		66	56	
Time since diagnosis,						
N	N = 1941	N = 1156	0.0330	N = 176	N = 2921	0.6199
Mean years	6.2	5.7		5.8	6.0	
Mobility problems (PRF-reported), mean	N = 2124	N = 1381		N = 181	N = 3324	
Paraesis	0.90	0.86	0.2499	0.90	0.88	0.5122
Plegia	0.25	0.26	0.9536	0.25	0.25	0.9063
Spasticity	0.59	0.48	0.0001	0.61	0.54	0.1503
Muscle atrophy	0.15	0.09	0.0001	0.03	0.14	0.0085
Spasms/cramps	0.29	0.22	0.0001	0.30	0.26	0.2680
Footdrop	0.15	0.08	0.0001	0.13	0.12	0.4241
Ataxia	0.58	0.53	0.1016	0.54	0.56	0.9508
Dystonia	0.04	0.02	0.0091	0.04	0.03	0.4537
Fatigue	0.84	0.77	0.0219	0.85	0.81	0.3428
Most recent EDSS score						
N	N = 1845	N = 1211	0.5724	N = 151	N = 2905	0.4510
Mean	3.24	3.20		3.1	3.2	

The distribution of patients by gender and employment type between the cohort who completed a PSC and those who did not was similar although there were proportionately more females and manual workers in the cohort who did not complete a PSC. The cohort who did complete a PSC tended towards slightly higher physician-reported mean scores across a range of mobility problems collected in the PRF although there was no difference in term so the most recent mean EDSS score. Few differences were noted between the cohort of patients for whom a T25FW score was available and those without such a score. The only significant difference was a higher physician-reported mean muscle atrophy score in the cohort with a T25FW score.

## Discussion

The results of the cross-sectional, patient record-based study presented here demonstrated that a significant proportion of patients with MS experience walking and mobility problems (WMPs) and the majority of patients consider them to be their most bothersome symptom. These data are consistent with those reported by Heesen and co-workers [26] who found that among a cohort of 166 patients with MS, walking was regarded by patients as the most valuable function, followed by visual functioning and cognition. These data add further weight to the importance of impaired walking ability as a core symptom from the patient’s perspective and therefore as a target for therapeutic intervention.

The results presented here also demonstrate a clear, independent, and strong directional relationship between the severity of WMPs (both from a subjective symptom assessment and from objective T25FW test results) and healthcare resource utilization (including the requirement for both professional and non-professional care), and assistance with walking. These data highlight the economic burden imposed by WMPs on MS patients. As such, these patients and/or healthcare systems will need to meet the costs of health and social care provision to address their daily needs. These results are consistent with a recent European survey in which patients were stratified based on their EDSS score [8]. The EDSS relies heavily on walking ability as a component of overall disease-related disability, and Kobelt and co-workers reported that requirements for, and costs associated with, healthcare resource utilization, the need for informal care, and home-based modification and aids increased with increasing EDSS-assessed disability.

Work activity was also markedly impacted by the presence of WMPs. Even patients who reported mild WMPs experienced some impairment in terms of work activity suggesting a societal need for addressing this functional impairment early in the disease course. These data are consistent with those reported by Kobelt and co-workers ([8] and also Salter and co-workers [11] and are of particular relevance given that patients are often diagnosed with the disease during early adulthood. In the European survey reported by Kobelt and co-workers (described above), work capacity (proportion of patients ≤65 years working) declined as EDSS-assessed disability increased. Salter and co-workers [11] used the NARCOMS mobility performance sub-scale to examine the impact of reduced mobility on activities of daily living and socioeconomic status. As observed in the current study, decreasing mobility was associated with increased unemployment and decreased full-tome work status, even at low levels of reduced mobility. Such early and on-going impact on ability to work has the potential to impact on a patient’s long-term quality of life and psychological well-being.

The DSP represents a large cross-sectional study of patients with MS of all severities and the physicians treating them. The data generated provides an insight into the problems associated with MS and the social and economic burden presented by this debilitating disease. Limitations specific to the MS DSP include the sample size in relation to the T25FW with data available for 184 patients (5.1% of the total study population). However the T25FW is a measure which, while used in clinical studies, is not routinely used in clinical practice. In the DSP sample, the 184 patients for whom T25FW was available were provided by 62 of the 340 physicians who participated; 278 physicians did not provide any T25FW data. Physicians were not asked to conduct this evaluation specifically for the purposes of this study but to provide the data where previously available in the medical record or where the test had been conducted as part the routine consultation process. An additional analysis did not identify any consistent differences between the cohort of patients for whom T25FW data were available and those without such data. This observation suggests that the subset of patients for whom T25FW data were available was representative of the whole cohort in terms of the severity of a range of mobility issues as rated by their physicians. Some differences were noted between the cohort of patients who completed a PSC and those who did not. For example, in the cohort of patients who did complete a PSC the proportion of females was slightly lower and the proportion engaged in manual work was somewhat higher. In addition, physician ratings of mobility problems were generally higher among those who completed a PSC. This latter observation suggests that the cohort with PSC data may have had more severe mobility issues although the actual numerical differences in the mean scores were small. The population studied here represents the population of MS patients consulting a neurologist and so may exclude patients not directly or routinely under neurologist care at the time of the study. Thus the population represented here are patients more likely to be receiving a degree of routine specialist MS care.

## Conclusion

In conclusion, while the results presented should be considered within the context of the limitations regarding their generalizability to the whole population of patients with MS, they do provide a valuable insight into the potential for significant impact of walking and mobility issues on all aspects of a patients life. Consequently, the results also highlight the need for clinicians to question patients about their walking and mobility problems at the first encounter and to monitor the patient for any changes at regular intervals (at least at each clinic visit). Finally, this Europe-based survey of the burden of mobility problems among patients with MS demonstrated that these patients could benefit from therapies that specifically address functional impairment of walking and mobility. Therapies that specifically improve WMPs including walking speed as measured, for example, using the T25FW, could have a positive socio-economic impact by potentially reducing healthcare resource utilization, the requirement for both formal and informal care, and mobility aids, as well as improving or preserving work capacity.

## Competing interests

The authors declare that they have no competing interests; all authors were employees of either Adelphi Real World or Biogen Idec at the time the research was conducted. The Adelphi MS Disease Specific Programme is an independent study owned by Adelphi, however analysis for this manuscript was funded by Biogen Idec.

## Authors’ contributions

EJ contributed to the design and conception of the Disease Specific Programme. JPe conducted all the statistical analyses. EJ, JPy, JPe, PA, KR all contributed to the interpretation of the data; drafting the article, revising it critically for intellectual content and final approval of the version to be published. All authors read and approved the final manuscript.

## Pre-publication history

The pre-publication history for this paper can be accessed here:

http://www.biomedcentral.com/1471-2377/12/94/prepub
